# Transactions of the Medical Society of the State of New York, at Its Annual Meeting in the City of Albany, Held February, 1854

**Published:** 1853

**Authors:** 


Art. XVII.—*Transactions of the Medical Society of the State of New York, at its Annual Meeting in the City of Albany, held February,* 1854. Albany: Van Benthuysen. 1854.

This tract is published by the State of New York, as among the doings of its Assembly. New York does things on a scale worthy of the intelligence and greatness of the *Empire* State. When a member of the general assembly of Kentucky was requested at the last session to apply for an appropriation to publish the transactions of the Kentucky Medical Society, he replied that he "should be laughed at if he were to make such a proposition, and that his motion would not receive a single vote!"

The Transactions of the State Medical Society of New York are made up chiefly of the following papers:—

"1. Annual Address before the State Medical Society; by Jenks S. Sprague, M. D., President. 2. Extra-Uterine Conception, reported for the State Society; by Wm. H. H. Parkhurst. 3. Penetrating wounds of Abdomen, Punctured wounds of Intestines, and Penetrating wounds of the Larynx; by Alden March, M. D. 4. Dislocated Femur—Reduction; by Samuel Shumway, M. D. 5. Annual Address before Albany Co. Medical Society; by J. V. P. Quackenbush, M. D., on Prolapsus of the Bladder, with Biographical sketches of the late Doctors Lewis C. Beck and David Martin. 6. Annual Address before Onondaga Co. Medical Society; by Jonathan Kneeland, M. D., President. 7. Rational Medicine, or the regular Practice as it is: by Merrit H. Cash, M. D. 8. Biographical Sketch of the late Henry W. Doolittle, of Herkimer. 9. Abstract of the Proceedings of the Medical Society of the State of New York, at its Annual Meeting, 1854."

